# Zeb2 is a negative regulator of midbrain dopaminergic axon growth and target innervation

**DOI:** 10.1038/s41598-017-08900-3

**Published:** 2017-08-17

**Authors:** Shane V. Hegarty, Sean L. Wyatt, Laura Howard, Elke Stappers, Danny Huylebroeck, Aideen M. Sullivan, Gerard W. O’Keeffe

**Affiliations:** 1 Department of Anatomy & Neuroscience, University College Cork (UCC), Cork, Ireland; 2 School of Biosciences, Cardiff University, Museum Avenue, Cardiff, CF10 3AT UK; 3 Department of Development and Regeneration, Laboratory of Molecular Biology (Celgen), KU Leuven, 3000 Leuven, Belgium; 4 Department of Cell Biology, Erasmus University Medical Center, 3015 CN Rotterdam, The Netherlands; 5 APC Microbiome Institute, UCC, Cork, Ireland; 6 The INFANT Centre, CUMH and UCC, Cork, Ireland

## Abstract

Neural connectivity requires neuronal differentiation, axon growth, and precise target innervation. Midbrain dopaminergic neurons project via the nigrostriatal pathway to the striatum to regulate voluntary movement. While the specification and differentiation of these neurons have been extensively studied, the molecular mechanisms that regulate midbrain dopaminergic axon growth and target innervation are less clear. Here we show that the transcription factor Zeb2 cell-autonomously represses Smad signalling to limit midbrain dopaminergic axon growth and target innervation. Zeb2 levels are downregulated in the embryonic rodent midbrain during the period of dopaminergic axon growth, when BMP pathway components are upregulated. Experimental knockdown of Zeb2 leads to an increase in BMP-Smad-dependent axon growth. Consequently there is dopaminergic hyperinnervation of the striatum, without an increase in the numbers of midbrain dopaminergic neurons, in conditional *Zeb2* (*Nestin*-Cre based) knockout mice. Therefore, these findings reveal a new mechanism for the regulation of midbrain dopaminergic axon growth during central nervous system development.

## Introduction

The establishment of neural pathways in the developing central nervous system (CNS) requires coordinated regulation of neuronal differentiation, axon growth and target innervation^1^. The signals that guide catecholaminergic innervation have been intensively studied in the peripheral nervous system (PNS), where the neurotrophic theory states that limiting amounts of target-derived neurotrophic factors maintain the survival of the required numbers of neurons, and promote the growth of innervating axons in their targets^2, 3^. More recently, it has emerged that cell-autonomous signalling operates in these neurons to regulate axon growth and target innervation^4–6^. In the CNS, most catecholaminergic neurons are found in the brainstem, of which one of the largest populations are the midbrain dopaminergic (mDA) neurons of the substantia nigra that project axons to the striatum. Nigrostriatal mDA neurons are generated between embryonic day (E) 10 and E12 in mice (E12-E14 in rat)^7^. mDA axons extend rostrally from E11, reach the striatum at E12, and innervate the rostral part of striatum by E16 in mice (E18 in rat)^8^. Naturally-occurring cell death occurs in the postnatal period (~P0-P20) to prune the initially overproduced nigrostriatal mDA neurons, and depends on target-derived neurotrophic factors such as GDNF^9–11^. The nigrostriatal pathway is crucial for voluntary motor control^12^, as highlighted by the progressive loss of striatal mDA innervation in Parkinson’s disease (PD)^13^. While the molecular basis of mDA specification and differentiation has been extensively characterised^7^, it remains largely unknown whether cell-autonomous mechanisms play a role in regulating axon growth and target innervation of mDA neurons in the CNS.

From a PCR screen to identify new regulators of mDA axon growth and innervation, we detected the expression of *Zinc finger E-box-binding homeobox 2* (*Zeb2*) mRNA in the midbrain during the period of striatal innervation. Zeb2 (Smad-interacting protein-1; Sip1; and Zfhx1b) was identified as a DNA-binding transcriptional repressor that interacts with activated Smad1^14^, a mediator of bone morphogenetic protein (BMP) signalling. Within the embryonic CNS, Zeb2 controls hippocampal neuron development and is crucial for the correct timing of neurogenesis and gliogenesis in the neocortex in a non-cell-autonomous manner^15, 16^. Zeb2 also acts cell-autonomously to control: guided migration of cortical GABAergic interneurons^17, 18^; neocortical axon growth^19^; myelinogenesis in the CNS^20^; and (re)myelination in the PNS^21, 22^. In humans, mutations in one *ZEB2* allele causes Mowat-Wilson syndrome (MOWS, OMIM#235730), which includes severe neurodevelopmental defects^23, 24^. Here we investigate for the first time the role of Zeb2 in developing mDA neurons. We describe a new function for Zeb2 as a negative regulator of mDA axon growth and target innervation.

## Results

### Temporal-regulation of *Zeb2* expression, and of BMP pathway components, in developing midbrain *in vivo*

**Figure 1:**
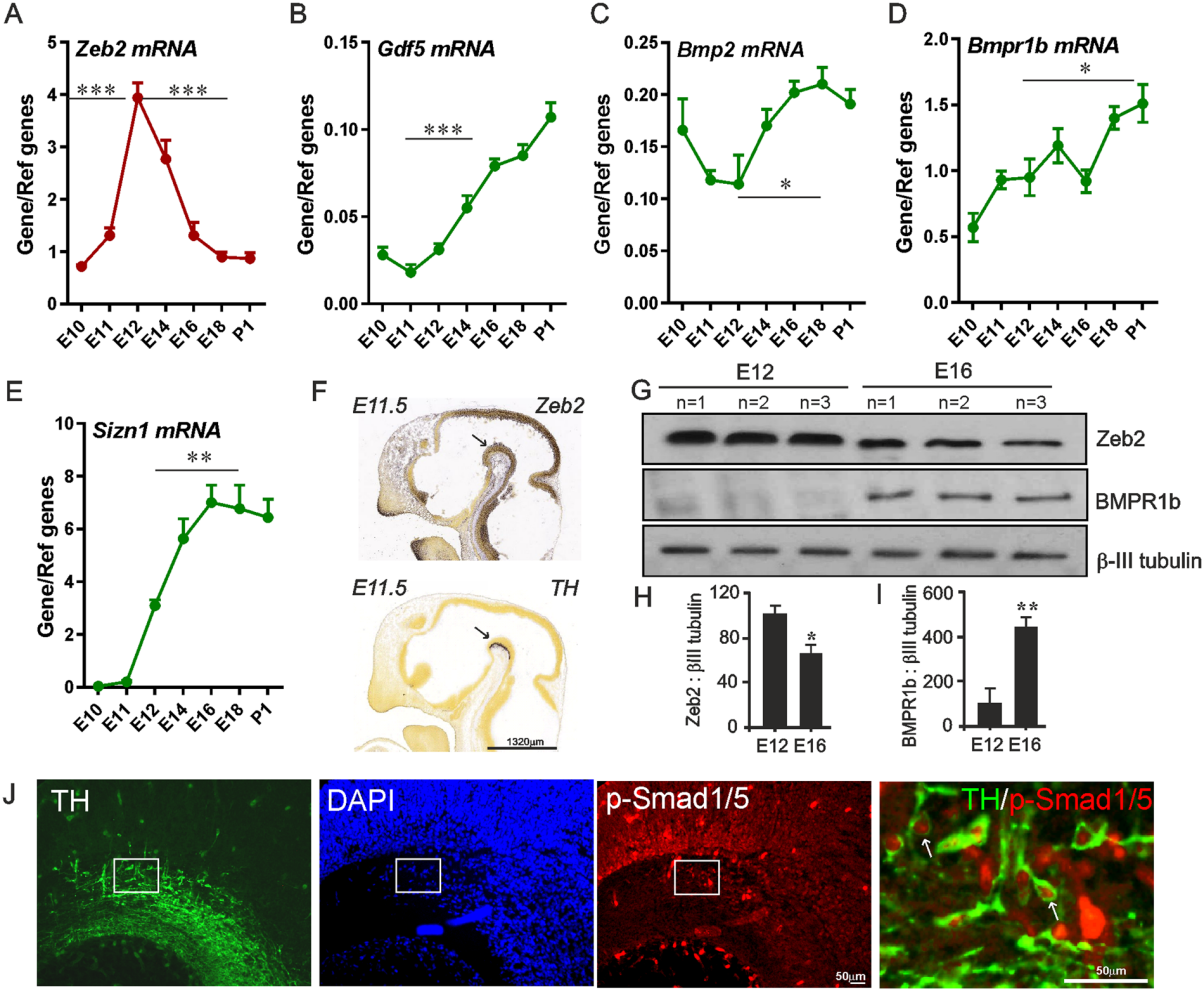
Temporal regulation of Zeb2 expression in the developing midbrain. (**A** to **E**) RT-qPCR of mRNA levels of (**A**) *Zeb2* (**B**) *Gdf5*, (**C**) *Bmp2*, (**D**) *Bmpr1b* and (**E**) *Sizn1* in the mouse midbrain from E11 to P1 relative to the levels of the geometric mean of three reference mRNAs *Gapdh*, *Sdha* and *Hprt1*. (**F**) *In situ* hybridisation of *Zeb2* and *TH* mRNA in sagittal sections of the E11.5 mouse VM (indicated by arrow) (©Allen Developing Mouse Brain Atlas, 2012). (**G**) Western blots of mouse midbrain protein extracts showing Zeb2 and BMPR1b protein levels at E12 and E16. (**H**) Quantification of Zeb2 and (**I**) BMPR1b protein levels in the mouse midbrain at E12 and E16. βIII tubulin was used as a loading control. (**J**) Immunohistochemistry showing p-Smad1/5 (red) co-localising with TH (green) in the E14 rat VM (indicated by arrow). (number of repetitions (**N**) = 3–6 from separate litters; mean ± SEM) (*p < 0.05, **p < 0.01, ***p < 0.001; E12 v E16 or E18; t-test or ANOVA with post hoc Tukey’s test). Scale bar as indicated.

Quantification of mRNA levels in the mouse ventral midbrain (VM) at intervals throughout the period of initial mDA axon growth and striatal innervation revealed significant differences in *Zeb2* expression during development. *Post-hoc* testing showed that *Zeb2* mRNA levels significantly increase from E10 to peak at E12, followed by a sharp decrease from E12 to E18 (p < 0.0001; Fig. 1A). Because Zeb2 represses BMP signalling during nervous system development^25^, we next investigated the expression of genes encoding components of the BMP pathway, which are subject to synexpression, autoregulation and feed-forward/-back regulation^14, 25–27^. We hypothesised that *Zeb2* downregulation may be paralleled by modulated expression of some of these BMP pathway components. To test this, we quantified transcript levels of *Gdf5, Bmp2, Bmpr1b* and *Sizn1* over the same time range. Significant age-dependent changes in expression were observed for *Gdf5*, *Bmp2*, *Bmpr1b* and *Sizn1* (which encodes a transcriptional co-activator downstream of BMP signalling). These displayed different profiles to that of *Zeb2*, significantly increasing from E12 to E18/P1 (p < 0.05; Fig. 1B to E). Such changes in *Zeb2* and *Bmpr1b* mRNA expression were also observed in the rat VM between E14 and E18 (data not shown and^28^). *In situ* hybridisation confirmed the quantitative real-time PCR (RT-qPCR) data, and showed localisation of *Zeb2* transcripts in the E11.5 mouse VM (Fig. 1F). Moreover, western blot was also used to demonstrate that Zeb2 and BMPR1b protein levels display these age-dependent expression changes in the mouse VM between E12 and E16 (Fig. 1G to I). Finally, immunohistochemistry was used to examine phospho-Smad1/5 signalling in the VM at the time of mDA axogenesis. Triple^**_**^labelled preparations in which mDA neurons were identified by tyrosine hydroxylase (TH) staining revealed that p-Smad1/5 were present in, but not limited to, mDA neurons in the E14 rat VM (Fig. 1J). This indicates that BMP-Smad signalling is active during mDA axonal growth in the developing midbrain.

### Zeb2 is a cell-autonomous negative regulator of BMP-Smad1/5 signalling in cultured midbrain neurons

**Figure 2:**
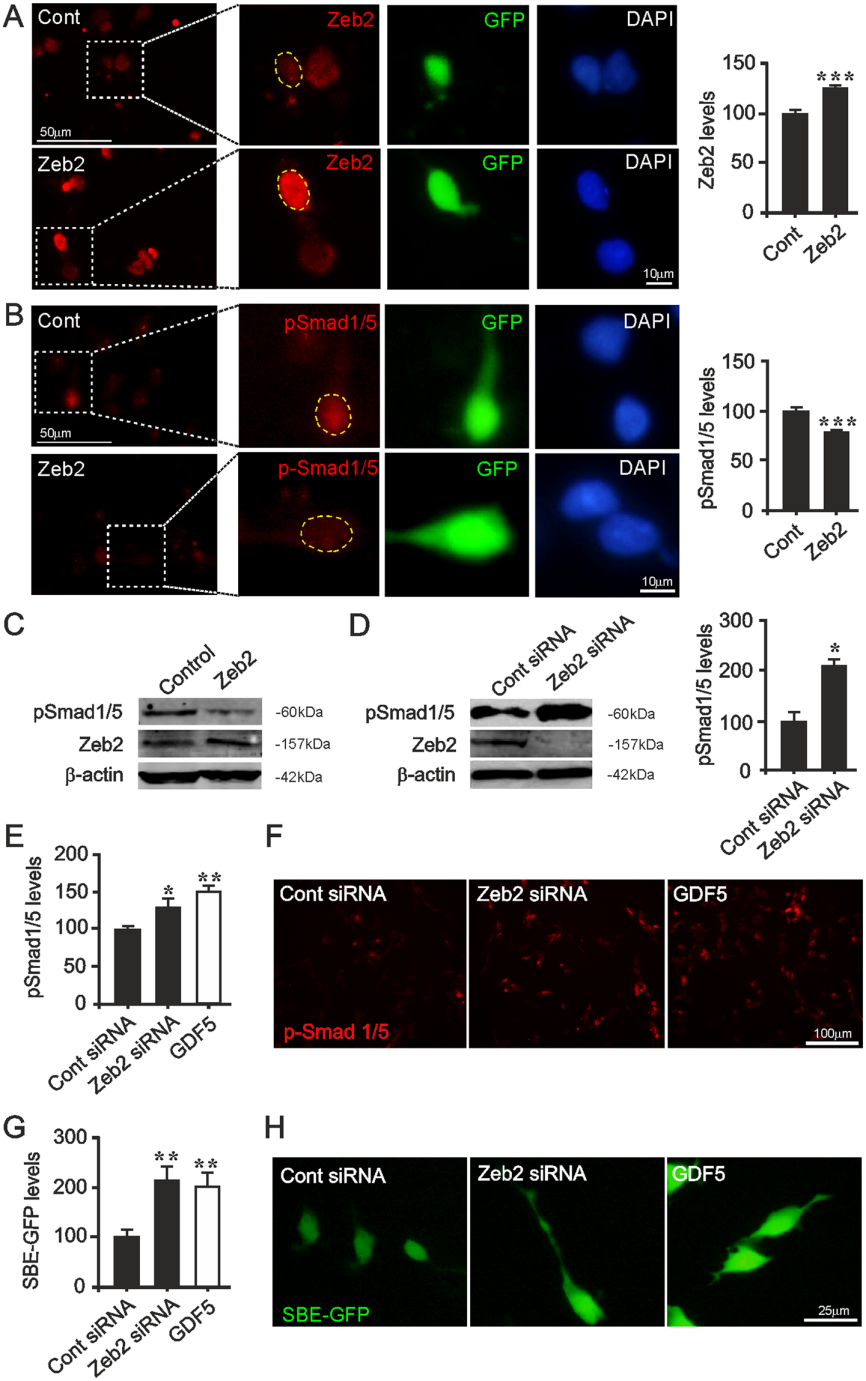
Zeb2 regulates p-Smad1/5 levels and p-Smad-dependent transcription. (**A**) Primary cultures of E14 rat VM neurons, transfected with a GFP expression vector and either a Zeb2 or control (Cont) expression vector, confirmed by immunocytochemistry with densitometry in GFP + cells at 24 h. (**B**, **C**) p-Smad1/5 levels as determined by (**B**) immunocytochemistry and (**C**) western blots in SH-SY5Y cells, and with densitometry in GFP^+^ E14 VM rat neurons 24 h after transfection with control or *Zeb2* expression vector (**B**). (**D**, **E**) p-Smad1/5 levels as determined by (**D**) western blots in SH-SY5Y and (**E**) E14 rat VM cultures 48 h after transfection with control siRNA or *Zeb2*-targeting siRNA, or treated with 100 ng/ml GDF5. β-actin was used as a loading control. (**F**) Representative photomicrographs of p-Smad1/5 levels in cells treated with GDF5 or transfected with Zeb2 siRNA at 48 h. (**G**) Graph and (**H**) representative photomicrographs of the relative levels of SBE-GFP fluorescence in SH-SY5Y cells transfected with a SBE-GFP reporter vector with either a control siRNA or *Zeb2*-siRNA, or treated with 100 ng/ml GDF5 for 24 h. All data are mean + SEM. (n = 3; *P < 0.05, **P < 0.01, ***P < 0.001 vs. control; t-test or ANOVA with post hoc Tukey’s test). Scale bar as indicated.

To determine if Zeb2 functions as a repressor of p-Smad1/5 signalling in primary VM neurons, we co-transfected E14 rat VM cells with a green fluorescent protein (GFP)-vector and either a control or Zeb2 expression vector. The latter resulted in increased Zeb2 expression in transfected (GFP^+^) cells (p < 0.001; Fig. 2A). Upon vector-encoded *Zeb2* overexpression, p-Smad1/5 levels in E14 VM cells were significantly reduced at 24 hour (h) post-transfection (p < 0.001; Fig. 2B). We also used SH-SY5Y cells, which are often used to model DA neurons and in which BMP-Smad signalling promotes neurite growth^29, 30^, and found that Zeb2 overexpression reduced p-Smad1/5 levels (Fig. 2C).

We next transfected both cell types with scrambled or *Zeb2*-targeting siRNA, and found that knockdown of Zeb2 resulted in a significant increase in p-Smad1/5 levels in both SH-SY5Y and E14 VM cells (p < 0.05; Fig. 2D to F). Zeb2 knockdown increased p-Smad1/5 levels to levels comparable to those in cultures treated with 100 ng/ml growth differentiation factor 5 (GDF5/BMP14), which was used as a positive control. To determine if this upregulation of p-Smad1/5 levels altered Smad-dependent transcription, SH-SY5Y cells were co-transfected with a reporter plasmid in which GFP expression is driven by a promoter containing Smad-binding elements (SBE-GFP)^28, 31^, together with either scrambled or *Zeb2*-siRNA. Control cultures were transfected with the SBE-GFP reporter vector, and treated without or with GDF5, the latter resulting in p-Smad-dependent reporter transcription. Zeb2 knockdown also led to a significant increase in Smad-dependent transcription (indicated by GFP expression) at 48 h (p < 0.01; Fig. 2G and F). These data show that Zeb2 is a negative regulator of p-Smad1/5 signalling in VM cells, and suggest that decreased Zeb2 expression may facilitate Smad-promoted axon growth.

### Zeb2 downregulation is necessary and sufficient for Smad-promoted neurite growth

**Figure 3:**
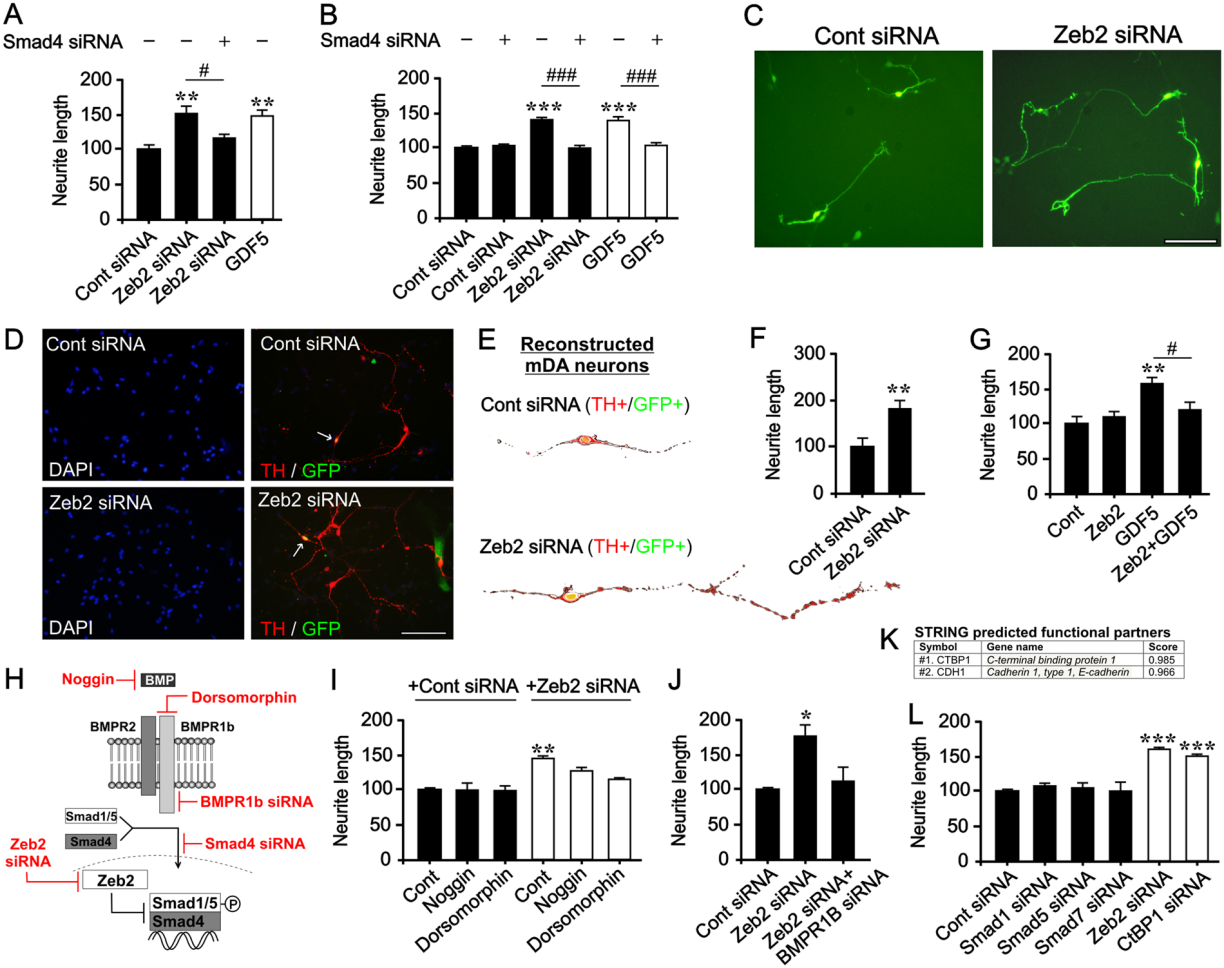
Zeb2 is an endogenous negative regulator of Smad-dependent neurite growth. (**A**,**B**) Graphs of neurite length relative to control in cultures of (**A**) SH-SY5Y and (**B**) E14 VM neurons transfected with *Zeb2*-siRNA or a control siRNA with or without a *Smad4*-siRNA and cultured for 72 h. Cells were treated with 100 ng/ml GDF5 as a positive control. (**C**, **D**) Photomicrographs of (**C**) GFP + neurons and (**D**) mDA neurons (TH + /GFP + ) in cultures of E14 rat VM neurons transfected with *Zeb2*-siRNA or a control siRNA at 72 h. (**E**) Reconstructed mDA neurons and (**F**) relative neurite length of mDA neurons from the experiment outlined in (**D**). (**G**) Graph of relative neurite length in cultures of E14 VM neurons transfected with *Zeb2* or control expression vectors cultured with or without 100 ng/ml GDF5 for 72 h. (**H**) Schematic of experimental approaches. (**I**,**J**) Graph of neurite length in cultures of E14 VM neurons transfected with *Zeb2*-siRNA or a control siRNA and cultured with noggin or dorsomorphin (**I**) or a control siRNA and *BMPR1b*-siRNA (**J**), after 72 h. (**K**) STRING score showing the two functional binding partners of Zeb2. (**L**) Graph of neurite length in cultures of E14 VM neurons transfected with control siRNA or *Smad1*, *Smad5*, *Smad7*, *Zeb2* or *CtBP1* siRNA and cultured for 72 h. (n = 3; *P < 0.05, **P < 0.01, ****P < 0.0001 vs. control; t-test or ANOVA with post hoc Tukey’s test; # *P* < 0.05; ### *P* < 0.001). All data are the mean + SEM. Scale bar = 100 µm.

To test this hypothesis, SH-SY5Y cells and E14 VM neurons were co-transfected with a GFP-vector and either a scrambled or *Zeb2*-siRNA, or treated with 100 ng/ml GDF5, and neurite growth was examined 72 h later. Knockdown of Zeb2 resulted in a significant increase in neurite growth in both cell types (p < 0.001; Fig. 3A to C). This increase in neurite growth was entirely prevented by Smad4 knockdown (Fig. 3A and B), which inhibits p-Smad-dependent transcriptional activity^28, 29^. To identify mDA neurons in GFP-transfected E14 VM cultures, cells were immunocytochemically stained for TH (Fig. 3D). Knockdown of Zeb2 resulted in a significant increase in neurite length in TH + (and GFP + ) mDA neurons at 72 h (p < 0.01; Fig. 3E and F). In contrast, vector-overexpressed Zeb2 did not affect basal levels of neurite growth in E14 VM neurons, whereas it completely prevented GDF5-promoted neurite growth (p < 0.05; Fig. 3G). We next examined whether Zeb2 modulates GDNF-induced neurite growth of E14 VM neurons, and found that Zeb2 overexpression did not inhibit the growth-promoting effects of GDNF (data not shown). These results demonstrate that high levels of Zeb2 maximally repress BMP-Smad-induced neurite growth. They also show that Zeb2 represses p-Smad1/5 activity in the basal state below a neurite growth-promoting threshold, and that downregulation of Zeb2 expression in mDA neurons facilitates Smad-promoted neurite growth.

### BMPR signalling is necessary for axon growth induced by Zeb2 downregulation

We next determined whether the neurite growth promoted by Zeb2 downregulation involves extracellular and/or intracellular mechanisms. As the increase in neurite growth that occurred following RNAi-based knockdown of endogenous Zeb2, required Smad4 (Fig. 3A and B), we focused on the canonical BMP-Smad signalling pathway. E14 VM neurons were co-transfected with a GFP vector and either a scrambled or *Zeb2*-siRNA, and then cultured with or without noggin (a secreted BMP antagonist) or dorsomorphin (a BMP receptor (BMPR) type 1 inhibitor) for 72 h (Fig. 3H). While knockdown of Zeb2 promoted a significant increase in neurite length (p < 0.01), noggin or dorsomorphin-mediated inhibition of BMPR activation significantly inhibited *Zeb2*-siRNA-induced neurite growth (Fig. 3I). We subsequently tested whether BMPR1b was required for neurite growth following Zeb2 downregulation by transfecting *Zeb2*-siRNA-treated VM cells with *BMPR1b-*siRNA^28, 29^, and found that this completely prevented neurite growth (p < 0.05; Fig. 3J).

We hypothesised that if the mechanism involved in Zeb2-mediated repression of p-Smad-dependent neurite growth occurs at the transcriptional level, then knockdown of a key transcriptional co-factor and binding partner of Zeb2 may also increase neurite length. To investigate this, we used STRING analysis to identify the top predicted functional binding partner of Zeb2, which was C-terminal binding protein 1 (CtBP1) (Fig. 3J)^26, 32^. Knockdown of CtBP1 in E14 VM neurons had the same neurite growth-promoting effect as knockdown of Zeb2 (p < 0.001; Fig. 3L), an effect also observed in SH-SY5Y cells (data not shown). This suggests that a Zeb2-CtBP transcriptional repressor complex acts to inhibit mDA axonal growth. Interestingly, treatment with noggin or dorsomorphin (Fig. 3I), or knockdown of Smad1, Smad5, or Smad7 (an inhibitory Smad that negatively regulates BMP-Smad signalling) (Fig. 3L), had no effect on neurite length. This further supports the conclusion that basal levels of Zeb2 repress p-Smad signalling below a growth-promoting threshold. Taking these findings together, Zeb2 appears to regulate mDA neuronal growth through a BMPR-Smad1/5 dependent mechanism, raising the possibility that Zeb2 may also act to control the extent of target innervation *in vivo*.

### *Zeb2* knockout mice have dopaminergic hyperinnervation of the developing striatum

**Figure 4:**
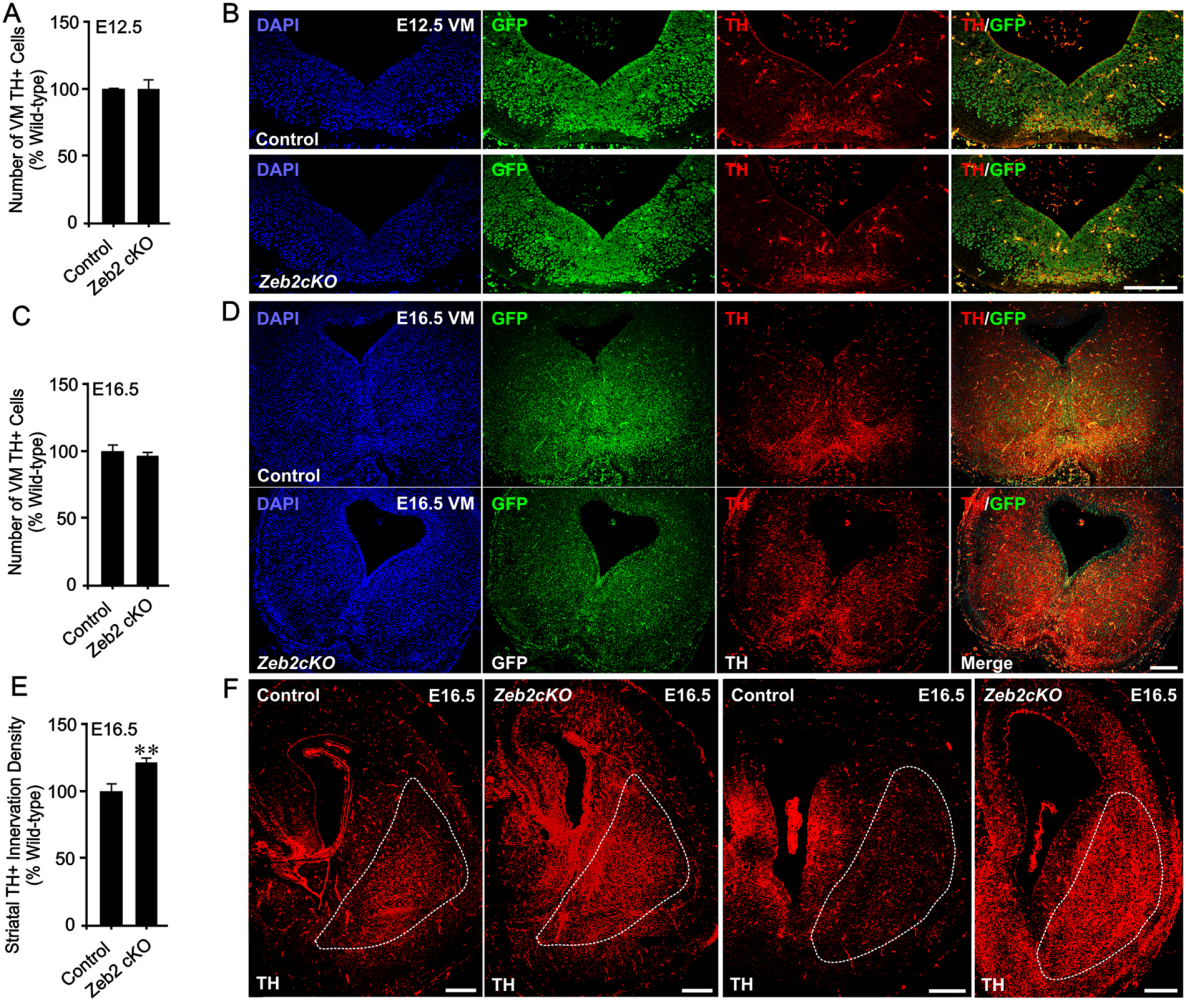
Zeb2-deficient mice have dopaminergic hyperinnervation of the striatum. (**A**,**C**) Graph of mDA neuron number in *Zeb2 cKO* mice and mice expressing normal levels of *Zeb2* (control) at (**A**) E12.5 and (**C**) E16.5. (**B**, **D**) Representative sections of the midbrain *Zeb2 cKO* and control mice at (**B**) E12.5 and (**D**) E16.5 showing DAPI (blue), GFP (green) and TH (red). (**E**) Graph of TH + striatal innervation density at E16.5 in *Zeb2 cKO* and Control mice. (**F**) Representative TH-stained coronal sections through the striatum of *Zeb2 cKO* and control mice at E16.5. The representative serial sections were taken at positions 160 µm and 280 µm through the striatum respectively (left hemispheres shown). (n = 5; **p < 0.01 vs. control; t-test). All data are the mean + SEM. Scale bar = 100 µm.

To assess the physiological relevance of Zeb2 in mDA axon growth *in vivo*, we quantified the numbers of mDA neurons, and DA innervation density of the developing striatum, in conditional *Zeb2*-deficient mice (*Nestin*-Cre:*Zeb2* ^*fl/KO*^; *Zeb2 cKO*), and in mice previously demonstrated to produce normal levels of *Zeb2* (*Nestin-*Cre:*Zeb2* ^*fl/WT*^; control)^16^. There were no significant differences in the numbers of TH^+^ mDA neurons between the groups at E12.5 (Fig. 4A and B) or E16.5 (Fig. 4C and D). In the mouse, mDA axons extend from E11, reach striatum from E12 and innervate the rostral part of striatum by E16 (for review, see^7^). Therefore, we assessed striatal mDA innervation density by quantifying TH^+^ fibres in the striatum at E16.5, a stage at which mDA innervation of the striatum is well underway. TH^+^ innervation density in the striatum of E16.5 *Zeb2 cKO* was significantly greater than in controls (p < 0.01; Fig. 4E and F). To rule out the possibility that this increase in TH^+^ innervation density was due to increased TH expression, rather than increased DA innervation, we analysed the expression of TH following *Zeb2* knockdown *in vitro* and *in vivo*. We found no differences in TH expression in mDA neurons following *Zeb2* knockdown (data not shown). These findings suggest that Zeb2 plays a physiological role in regulating mDA axon growth and target innervation *in vivo*.

## Discussion

**Figure 5:**
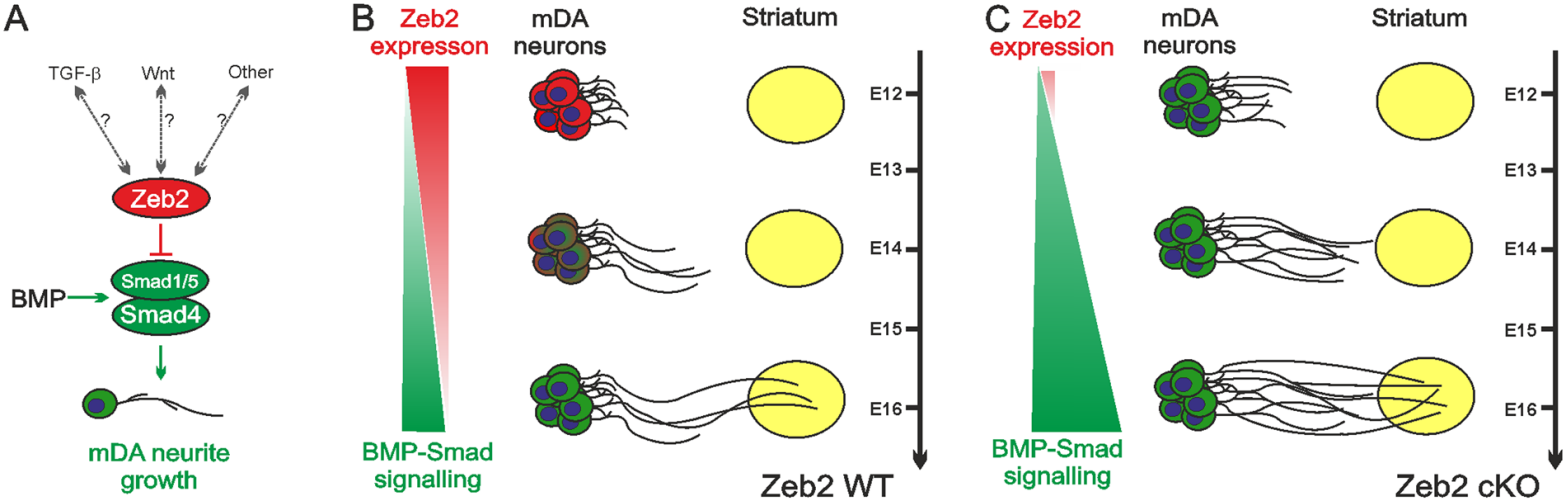
Zeb2 cell-autonomously represses Smad signalling to limit mDA neuronal growth and target innervation. (**A**) Schema showing the proposed role for Zeb2 as a cell-autonomous regulator of BMP-Smad1/5 signalling, which promotes neurite growth in mDA neurons. In future work it will be interesting to disentangle any potential cross-talk between Zeb2 and other molecular pathways that promote neurite growth in mDA neurons, including TGF-βs and Wnts. (**B**) Proposed model of the contribution of Zeb2 to the embryonic development of mDA target innervation based on current data. During peak mDA neurogenesis at ~E12 in mice, high levels of Zeb2 repress BMP-Smad signalling to limit mDA neurite growth. As mDA neurons extend axons and innervate the striatum from E12-E16, a reduction in Zeb2 levels leads to an increase in BMP-Smad-dependent transcriptional activity which promotes neurite growth in mDA neurons. (**C**) Knockdown of Zeb2 removes a cell-autonomous inhibitory mechanism that results in excessive mDA neurite growth through BMPR1b-Smad-dependent signalling, which causes hyperinnervation of the striatum *in vivo*. Whether this mDA hyperinnervation persists throughout the lifespan is an important topic for future investigation. In summary, these data provide new evidence for a Zeb2-controlled signalling mechanism that operates in mDA neurons to regulate axon growth and target innervation during embryonic CNS development.

We report that Zeb2 is a novel negative regulator of mDA axon growth and target innervation during embryogenesis (Fig. 5). Downregulation of Zeb2 expression in embryonic VM neurons resulted in promotion of axon growth via Smad signalling, while its absence in *Zeb2*-deficient mice led to mDA striatal hyperinnervation. In the mouse VM, *Zeb2* expression increased from E10 to sharply peak at E12, suggesting that Zeb2 may contribute to mDA neurogenesis^33^, as it does in the hippocampal anlage and the embryonic neocortex^15, 16^. However, no changes in numbers of TH^+^ mDA neurons were observed in the *Zeb2 cKO* mice at E12.5 or E16.5. Interestingly, *Wnt5a* is expressed in the mouse VM at E11.5 and is important for the initiation of neurite outgrowth, but by E14.5 causes neurite retraction^34^. Zeb2 has been identified as a candidate downregulator (either directly or indirectly) of *Sfrp1*, which encodes a secreted Wnt antagonist^15, 33^. Thus, high levels of Zeb2 at E12 may facilitate Wnt-promoted initiation of mDA axon growth, which may then become more responsive to BMP-Smad signalling-induction as *Zeb2* expression declines from E12-E18, due to a combination of increasing inhibition of Wnt signalling (via increased Sfrp1) and decreasing Zeb2-mediated inhibition of BMP-Smad signalling. Indeed, axonal growth requires integration of multiple signals, as well as the changing of responses to cues along their trajectory to targets^1^. This proposed mechanism is supported by our finding that Zeb2 negatively regulates BMP-Smad dependent gene transcription, in agreement with other studies^14, 20, 25^. Moreover, Zeb2 regulates Wnt and BMP signalling output during neural crest development, via a mechanism that may depend on Smads^25, 35^. In addition to this, TGF-β superfamily members, such as GDNF and TGF-βs, are expressed during nigrostriatal pathway development and regulate its development^9–11, 36–38^. While we found that Zeb2 did not inhibit the neurite growth-promoting effects of GDNF *in vitro*, it would be interesting in future work to disentangle any potential cross-talk between Zeb2 and other molecular pathways that promote neurite growth in mDA neurons, such TGF-βs and Wnts.

Downregulation of Zeb2 also facilitated axon growth through a BMP-BMPR-Smad mechanism. In support of this, canonical BMP-BMPR1b-Smad signalling induces mDA neuronal growth^28^. Furthermore, we found that increased Zeb2 levels represses GDF5-promoted, BMPR-Smad-mediated neurite growth. Our findings also suggest that such repression by Zeb2 is dependent on the CtBP co-repressor, similar to Zeb2-mediated neural induction in the amphibian embryo^26^. These data indicate that downregulation of Zeb2 during mDA axonal growth and target innervation may facilitate BMP-Smad-mediated induction of this developmental process. In support of this, BMPs and BMPRs are present in both the midbrain and striatum, as well as in mDA neurons, at key time-points for nigrostriatal pathway development^28, 36^. Thus, BMPs may act locally in the midbrain, and/or in the striatum as target-derived neurotrophic factors, to induce mDA axonal growth and target innervation in a process that, at least in part, is negatively regulated by Zeb2 levels in a cell autonmous manner. Indeed, axon growth and target innervation of peripheral catecholaminergic neurons is controlled by cell-autonomous signalling^4–6^, and is also regulated by Wnt5a and BMP-Smad signalling^6, 31^. In future studies, it will be important to further characterise the role of BMP ligands in mDA innervation, and to determine whether Zeb2 functions in a similar manner in peripheral catecholaminergic neurons.

We also report for the first time mDA hyperinnervation of the striatum in the *Zeb2 cKO* mouse embryos at E16.5. This may recapitulate some developmental defects in MOWS, in particular the enlarged basal ganglia^24^. However, whether this mDA hyperinnervation persists throughout the lifespan is an important topic for future investigation. Nigrostriatal mDA neurons are initially overproduced during neurogenesis, and subsequently undergo naturally-occurring cell death in the postnatal period (~P0-P20)^9^. Future studies should analyse striatal mDA fibre density and mDA neuronal number in the *Zeb2 cKO* mouse at ages following this period of postnatal developmental programmed cell death. Finally, progressive degeneration of striatal mDA innervation causes debilitating motor dysfunctions in PD^13^. There is robust evidence that mDA axonal degeneration in the striatum occurs early in PD pathogenesis, and precedes neuronal loss^39, 40^. Thus, transcription factors that control mDA axon growth may be novel candidate targets in future therapeutic approaches for PD^41^. In this regard, the present study suggests that Zeb2 downregulation may be a viable neurotrophic strategy to restore striatal mDA innervation for the treatment of PD. In summary, our work provides new evidence for a Zeb2-controlled signalling mechanism that operates in central catecholaminergic neurons to regulate axon growth and appropriate target innervation during embryonic development.

## Methods

### Cell Culture

All experiments were carried out in accordance with relevant guidelines and regulations, in compliance with law, and approved by the UCC Animal Ethics Experimentation Committee and the Health Products Regulatory Authority. For E14 rat VM cultures, E14 embryos were obtained by laporatomy from time-mated female Sprague-Dawley rats following decapitation under terminal anaesthesia induced by the inhalation of isoflurane (Isoflo®). Dissected VM tissue was centrifuged at 100 g for 5 minutes at room temperature. The tissue pellet was then incubated in a 0.1% trypsin-Hank’s Balanced Salt solution for 5 minutes, at 37 °C with 5% CO2. Fetal calf serum (FCS; Sigma) was then added to the tissue followed by centrifugation at 100 g for 5 minutes at 4 °C. The resulting cell pellet was resuspended in 1 ml of differentiation media (Dulbecco’s modified Eagle’s medium/F12, 33 mM D-glucose, 1% L-glutamine, 1% FCS, supplemented with 2% B27; all Sigma) using a P1000 Gilson pipette, and then carefully triturated using a sterile plugged flame-polished Pasteur pipette, followed by a 25-gauge needle and syringe. Cell density was estimated using a haemocytometer. Cells were plated on poly-D-lysine (Sigma)-coated 24-well tissue culture plates at a density of 5 × 10^4^ cells per well in 500 μl of differentiation media at 37 °C with 5% CO_2_.

SH-SY5Y cells (Sigma; passages 18–25) were used as a model of human DA neurons in this study. These were maintained in Dulbecco’s Modified Eagle Medium Nutrient Mixture F-12 (Sigma), supplemented with 10% foetal calf serum (Sigma), 100 nM L-Glutamine (Sigma), 100 U/ml Penicillin (Sigma), 10 µg/ml Streptomycin (Sigma), in a humidified atmosphere containing 5% CO_2_ at 37 °C.

Where indicated, cells were treated daily with 100 ng/ml of GDF5 (kindly provided by Biopharm GmbH), 1 μg/ml of dorsomorphin (Sigma) or 200 ng/ml of noggin (R&D Systems).

### Electroporation of Cultured Cells

Electroporation of cultured cells was carried out using the NeonTM Transfection System (Invitrogen). Cell suspensions were prepared for counting (as outlined above), and the required volume of cells to give 200,000 cells per well was centrifuged at 4 °C at 100 g for 5 minutes. The cell pellet was washed twice with 10 mM phosphate buffered saline (PBS) (without CaCl_2_ and MgCl_2_) (Sigma), and then resuspended in the required amount of the manufacturers resuspension buffer (12 µl per transfection/plasmid) (Invitrogen). 0.5 µg of *GFP* plasmid; 1 µg of desired plasmid DNA (*Zeb2* overexpression^42^ or *Smad4* siRNA^29^ vectors); and/or 500 nM of *Zeb2*, *CtBP1*, *Smad1*, *Smad5*, or *Smad7* siRNA (Life Technologies) was added to the resuspended cells. 10 µl of the cell/plasmid mixture was then electroporated according to the manufacturer’s protocol under specific parameters (1100 V; 30 ms; 2 pulses).

### Immunocytochemistry

Cultures were fixed for 10 minutes using 100% ice-cold methanol (E14 VM cells), or 4% paraformaldehyde (SH-SY5Y cells). Following 3 washes in 10 mM PBS-T (0.02% Triton X-100 in 10 mM PBS) for permeabilization, cultures were incubated in blocking solution (5% bovine serum albumin) for 1 h at room temperature. Cultures were subsequently incubated in the following primary antibodies: phopsho-Smad 1/5 (p-Smad1/5; 1:200; Cell Signalling), TH (1:200; mouse monoclonal; Millipore, or 1:300; rabbit polyclonal; Millipore), Zeb2 (1:50; Santa Cruz) and β-actin (1:200; Sigma) diluted in 1% bovine serum albumin in 10 mM PBS at 4 °C overnight. Following 3 × 5 minutes washes in PBS-T, cells were incubated in Alexa Fluor 488- and/or 594-conjugated secondary antibodies (1:500; Invitrogen) reactive to the species of the primary antibodies and diluted in 1% bovine serum albumin in 10 mM PBS, at room temperature for 2 h in the dark. Cultures were counterstained with bisbenzimide (1:1000 in 10 mM PBS; Sigma). Negative controls in which the primary antibody was omitted were also prepared (not shown). Cells were imaged under an Olympus IX70 inverted microscope fitted with an Olympus DP70 camera and AnalysisDTM software. For densitometric analysis, the fluorescence intensity of individual cells stained for p-Smad 1/5 was measured using Image J analysis software.

### Conditional Zeb2 knockout Mice

All animal experiments were carried out in compliance with law and were ethically approved. Mice carrying a floxed (exon 7) *Zeb2* allele (Zeb2fl/KO)^43^ were crossed with *Nestin*-Cre^44^ mice.

### Immunohistochemistry

Brains were dissected and washed in ice-cold PBS and fixed overnight with 4% paraformaldehyde (wt/vol) followed by progressive alcohol-assisted dehydration and paraffin embedding. 10-μm-thick serial sections were cut on a Leica microtome and then processed for immunohistochemistry using an automated platform (Ventana Discovery, Ventana Medical Systems) and mounted in DAPI-supplemented Mowiol. The following primary antibodies were used: p-Smad1/5 (1:50), TH (Novocastra; 1:500), and GFP (Novocastra; 1:500). For secondary antibodies, CY2-, CY3- or biotin-conjugated donkey antibodies (all at 1:600, Jackson ImmunoResearch) were used. Sections were photographed using a confocal radiance microscope connected to a spot camera (Visitron Systems) and data analysis was done with ImageJ software. 5 control and 5 knockout brains were analyzed, and 10 sections per brain were quantified. In each brain and time point, TH-positive cell bodies or TH-positive neurites/fibres were quantified in midbrain/striatum of the same coronal plane. TH-positive striatal fibres were analysed through 400 µm of the striatum, spanning from the rostral striatum to the region between the caudal striatum and diencephalon. Brain regions were identified using an atlas of the embryonic mouse brain and landmarks, such as the lateral ventricles, after which 10 µm serial sections (1:10 series) were counted and every fourth section was sampled from the striatum. For the striatal innervation experiments, TH-positive fibres were quantified in the E16.5 mouse striatum using ImageJ.

### Quantitative real-time PCR (RT-qPCR)

The midbrains of E10 to P1 CD-1 mice, obtained from time-mated pregnant dams as outlined above, were micro-dissected and total RNA was then extracted and purified. Midbrain samples were disrupted and homogenised in 1 ml of QIAzol Lysis Reagent (Qiagen). After the addition of 200 µl chloroform, homogenates were separated into aqueous and organic phases by centrifugation at 10,000 g for 15 minutes. The upper aqueous phase was mixed with an equal volume of 70% ethanol, to precipitate the RNA, and then transferred to an RNeasy Mini spin column placed in a 2 ml collection tube. Total RNA was purified using the Qiagen RNeasy Mini extraction kit and RNase-free DNase set, according to the manufacturer’s instructions.

The levels of *Zeb2*, *Gdf5*, *Bmp2*, *Bmpr1b* and *Sizn1* mRNAs were quantified by RT-qPCR relative to a geometric mean of mRNAs for the house keeping enzymes glyceraldehyde phosphate dehydrogenase (*Gapdh*), succinate dehydrogenase (*Sdha*) and hypoxanthine phosphoribosyltransferase-1 (*Hprt1*). 5 μl of total midbrain RNA was reverse transcribed for 1 h at 45 °C using the AffinityScript kit (Agilent, Berkshire, UK) in a 25 µl reaction according to the manufacturer’s instructions. 2 µl of cDNA was amplified in a 20 µl reaction volume using Brilliant III ultrafast qPCR master mix reagents (Agilent Technologies). PCR products were detected using dual-labelled (FAM/BHQ1) hybridization probes specific to each of the cDNAs (MWG/Eurofins, Ebersberg, Germany). The PCR primers were: *Zeb2* forward: 5′-GGT CCA GAA GAA ATG AAG-3′ and reverse: 5′-ATC TCT TTC CTC CAG TTT-3′; *Gdf5* forward: 5′-TAA TGA ACT CTA TGG ACC-3′ and reverse: 5′-GAT GAA GAG GAT GCT AAT-3′; *Bmp2* forward: 5′-CGT CAA GCC AAA CAC AAA-3′ and reverse: 5′-ACG ATC CAG TCA TTC CAC-3′; *Bmpr1b* forward: 5′-AGT GTA ATA AAG ACC TCC A-3′ and reverse: 5′-AAC TAC AGA CAG TCA CAG-3′; *Sizn1* forward: 5′- ACA ATG AGA GTG ACA AGG-3′ and reverse: 5′-GGT TAA TTT AAG GCT GGG-3′; *Gapdh* forward: 5′-GAG AAA CCT GCC AAG TAT G-3′ and reverse: 5′-GGA GTT GCT GTT GAA GTC-3′; *Sdha* forward: 5′-GGA ACA CTC CAA AAA CAG-3′ and reverse: 5′-CCA CAG CAT CAA ATT CAT-3′; *Hprt1* forward: 5′-TTA AGC AGT ACA GCC CCA AAA TG-3′ and reverse: 5′-AAG TCT GGC CTG TAT CCA ACA C-3′. Dual-labelled probes were: *Zeb2*: 5′-FAM-TGG CAA AGT ATT CCT CAA AGT CT-BHQ1-3′; *Gdf5*: 5′-FAM-TGA ATC CAC ACC ACC CAC TTG-BHQ1-3′; *Bmp2*: 5′-FAM-TCA CTG AAG TCC ACA TAC AAA GGG T-BHQ1-3′; *Bmpr1b*: 5′-FAM-CCA CTC TGC CTC CTC TCA AG-BHQ1-3′; *Sizn1*: 5′-FAM-TCG CAT CTT ACT GTG GTT CA-BHQ1-3′; *Gapdh*: 5′-FAM-AGA CAA CCT GGT CCT CAG TGT-BHQ1-3; *Sdha*: 5′-FAM-CCT GCG GCT TTC ACT TCT CT-BHQ1–3, *Hrpt1*: 5′-FAM-TCG AGA GGT CCT TTT CAC CAG CAA G-BHQ1–3′. Forward and reverse primers were used at a concentration of 150 nM and dual-labelled probes were used at a concentration of 300 nM. PCR was performed using the Mx3000 P platform (Agilent) using the following conditions: 95 °C for 3 minutes followed by 45 cycles of 95 °C for 12 seconds and 60 °C for 35 seconds. Standard curves were generated for each cDNA for every real time PCR run, by using serial threefold dilutions of reverse transcribed mouse adult brain total RNA (Zyagen, San Diego, USA). Relative mRNA levels were quantified in VMs dissected from 3–6 separate animals for each age. Primer and probe sequences were designed using Beacon Designer software (Premier Biosoft, Palo Alto, USA).

### Analysis of Neuronal Complexity

The total neurite length of individual cultured cells was measured at 72 h using Sholl analysis as previously described^45, 46^. Traces of GFP + /TH + neurons were carried out using the CorelDRAW × 4 software and analysed as previously described^47^. Briefly, neurite length (NL) was calculated using the following formula; NL = α x T x (π/2), where α is the number of times the neurite intersects the grid lines, and T is the distance between the gridlines on the magnified image (taking into account the magnification factor). Individual neurons with intact processes were analysed from within 20 random fields (1.6 × 10^5^ µm^2^) per condition, where any neuron with a process that was at least one and half times the length of the soma was determined as an intact process (which precludes analysis of apoptotic neurons).

### Western Blotting

Western blotting was carried out as described^48^. Cells or freshly dissected embryonic mouse VM tissue were lysed in radioimmunoprecipitation assay (RIPA) buffer (50 μl RIPA per 1 × 10^6^ cells or 5 mg of tissue; 50 mM Tris-HCL; 150 mM NaCl, 1% Triton X-100, 1 mM EDTA, 1 mM NaF, 1 mM Na3VO4, 1 μg/ml leupeptin and 1 μg/ml pepstatin) for 1 h on ice, and insoluble debris was removed by centrifugation. 15 μg of protein was run by SDS-PAGE and transferred to nitrocellulose membranes using a Mini Trans-Blot Electrophoretic Transfer Cell (Bio-Rad, CA, USA). The membranes were incubated with primary antibodies against p-Smad1/5 (1:1000), BMPR1b (1:1000; Abcam); Zeb2 (1:250), βIII tubulin (1:2000; R&D Systems) or β-actin (1:2000) overnight at 4 °C, washed, incubated with the appropriate goat IR700/800-labelled secondary antibodies (1:10000; Licor), washed and visualised with Odyssey (Licor). Protein levels were normalised to β-actin or βIII tubulin by densitometry using Image Studio Lite software (Licor).

### Statistical Analysis

Unpaired Student’s t-test or one-way ANOVA with a post hoc Tukey’s test was performed, as appropriate, to determine significant differences between groups. Results were expressed as means with SEM and deemed significant when p < 0.05.
